# Natural Killer Cells Offer Differential Protection From Leukemia in Chinese Southern Han

**DOI:** 10.3389/fimmu.2019.01646

**Published:** 2019-07-16

**Authors:** Zhihui Deng, Jun Zhao, Siqi Cai, Ying Qi, Qiong Yu, Maureen P. Martin, Xiaojiang Gao, Rui Chen, Jiacai Zhuo, Jianxin Zhen, Mingjie Zhang, Guobin Zhang, Liumei He, Hongyan Zou, Liang Lu, Weigang Zhu, Wenxu Hong, Mary Carrington, Paul J. Norman

**Affiliations:** ^1^Immunogenetics Laboratory, Shenzhen Blood Center, Shenzhen, China; ^2^School of Ophthalmology and Optometry, Shenzhen Eye Hospital, Shenzhen University, Shenzhen, China; ^3^Basic Science Program, Frederick National Laboratory for Cancer Research, Frederick, MD, United States; ^4^Department of Hematology, Shenzhen Second People's Hospital, Shenzhen, China; ^5^Central Laboratory, Baoan Maternal and Child Health Hospital, Shenzhen, China; ^6^Research and Development Department, Shenzhen Hank Bioengineering Institute, Shenzhen, China; ^7^Ragon Institute of MGH MIT and Harvard, Cambridge, MA, United States; ^8^Division of Biomedical Informatics and Personalized Medicine, Department of Microbiology and Immunology, University of Colorado Anschutz Medical Campus, Aurora, CO, United States

**Keywords:** leukemia, AML, ALL, CML, NHL, HLA, KIR, Chinese Southern Han

## Abstract

Interactions of human natural killer (NK) cell inhibitory receptors with polymorphic HLA-A, -B and -C molecules educate NK cells for immune surveillance against tumor cells. The *KIR A* haplotype encodes a distinctive set of HLA-specific NK cell inhibiting receptors having strong influence on immunity. We observed higher frequency of *KIR A* homozygosity among 745 healthy Chinese Southern Han than 836 adult patients representing three types of leukemia: ALL (OR = 0.68, 95% CI = 0.52–0.89, *p* = 0.004), AML (OR = 0.76, 95% CI = 0.59–0.98, *p* = 0.034), and CML (OR = 0.72 95% CI = 0.51–1.0, ns). We observed the same trend for NHL (OR = 0.47 95% CI = 0.26–0.88 *p* = 0.017). For ALL, the protective effect of the *KIR AA* genotype was greater in the presence of KIR ligands C1 (Pc = 0.01) and Bw4 (Pc = 0.001), which are tightly linked in East Asians. By contrast, the C2 ligand strengthened protection from CML (Pc = 0.004). NK cells isolated from *KIR AA* individuals were significantly more cytotoxic toward leukemic cells than those from other *KIR* genotypes (*p* < 0.0001). These data suggest KIR allotypes encoded by East Asian *KIR A* haplotypes are strongly inhibitory, arming NK cells to respond to leukemogenic cells having altered HLA expression. Thus, the study of populations with distinct *KIR* and *HLA* distributions enlightens understanding of immune mechanisms that significantly impact leukemia pathogenesis.

## Introduction

Natural killer (NK) cells can detect and eliminate leukemia cells (1). To perform this function, NK cells express multiple inhibitory and activating receptors specific for the HLA class I proteins that are expressed on tissue cells (2–4). Polymorphic inhibitory receptors educate NK cells, enabling them to kill tumors or infected cells having altered or reduced HLA class I expression. In turn, the activating receptors can complement this function by identifying foreign or neo-antigens (5–7). Study of NK cell receptor polymorphism is thus critical for understanding the anti-tumor effects of NK cells, and to facilitate their subsequent manipulation for use in immunotherapy (8).

Killer cell immunoglobulin-like receptors (KIR) are the most polymorphic of the human NK cell receptors, and are characterized by extreme variation in gene number (9). The *KIR* locus comprises up to 13 functional genes, *KIR2DL1-5, KIR2DS1-5, KIR3DL1/S1*, and *KIR3DL2-3*, where those designated with an “L” suffix encode inhibitory receptors and those with an “S” suffix encode activating receptors. Previous population, transplantation, and disease association studies have identified two functionally distinct groups of *KIR* haplotypes that are present in all populations (10, 11). *KIR A* haplotypes express four inhibitory receptors specific for polymorphic HLA class I (KIR2DL1, KIR2DL3, KIR3DL1, KIR3DL2), plus zero or one activating receptor (KIR2DS4) also specific for HLA class I. Multiple distinct *KIR B* haplotypes express fewer inhibitory receptors but more activating receptors than the *KIR A* haplotypes (12).

Although previous examinations of the role of KIR diversity in leukemia control have yielded potentially conflicting results (13), a common feature spanning multiple different populations and leukemia subtypes (14–19) is they are consistent with the *KIR A* haplotype being more frequent in healthy controls than in the patient groups. Because *AA* homozygous individuals possess all four inhibitory receptors and few activating receptors specific for HLA class I, their NK cells may therefore be strongly educated to detect any changes in HLA expression that can occur on leukemia cells (20). However, both the frequency of the *KIR A* haplotype, and the abundance of KIR ligands are highly variable across human populations (21, 22). In this context, examination of specific well-defined human populations is critical for understanding the role of KIR in leukemia control. Here we interrogate such effects in three leukemia subtypes sampled in a large cohort from Southern China, where the *KIR A* haplotype is very frequent.

## Materials and Methods

We studied 836 adult ALL, AML or CML patients, 225 pediatric leukemia patients and 745 healthy adult blood donors. We also studied 46 adult NHL patients, all having diffuse large B-cell lymphoma. The mean age of the adult patients analyzed was 31.4 years, controls 32.7 years, and pediatric cases 11.2 years. The patients were recruited from the hematopoietic stem cell transplantation (HSCT) program in the Shenzhen Blood Center, from August 1999 through June 2015. Leukemia diagnosis followed the French-American-British (FAB) classification, based on morphological observations. CML patients were not included if they had elected to take TKI (Imatinib) therapy instead of transplant. The CML patients included were from a study published in China (23). The controls were all unrelated healthy blood donors from Shenzhen Blood Center. All subjects are self-identified as Chinese Southern Han. Written informed consent was obtained from all study participants, and from the parents of all pediatric leukemia patients. The cohorts are described in Supplemental Figure 1.

### KIR and HLA Genotyping

The presence or absence of all 13 functional *KIR* genes and two pseudogenes was determined from genomic DNA using the PCR-SSP based *KIR* Ready Gene kit (Inno-Train Diagnostik GmbH, Kronberg Im Taunus, Germany). It is difficult to distinguish *KIR AB* heterozygotes from *KIR BB* homozygotes, but the *KIR AA* genotype is readily identifiable (9, 24). Thus, individuals were designated as homozygous for the *KIR A* haplotype if they tested positive for *KIR2DL1, KIR2DL3, KIR2DL4, KIR2DS4, KIR3DL1, KIR3DL2, KIR3DL3, KIR2DP1, KIR3DP1*, and no other *KIR*. *KIR2DL3* and *KIR3DL1/S1* alleles were determined by Sanger sequencing from a subset of 306 controls, as described (25–27). *HLA class I* genes were sequenced as previously described (28), and assigned genotypes at two field (distinct polypeptide sequence) resolution using the ASSIGN4.7 software (Conexio Genomics, Applecross, Australia).

### Statistical Analysis

For comparison of *KIR AA* frequencies, data from other populations were obtained from Allelefrequencies.net (21). The database was accessed in August 2018 and all populations having *KIR AA* genotype frequency data were used (*N* = 154). The populations were divided into major geographic origins as indicated (Amerindian, East Asian, European, Oceanian, sub-Saharan African) (21). The frequencies of *KIR AA* genotypes were compared across these major population groups using ANOVA, and, when comparing two populations, using unpaired *t*-tests for comparison of means. The tests were performed using GraphPad software (https://www.graphpad.com/). Odds ratio and confidence intervals were calculated using MedCalc (https://www.medcalc.org/calc/). Where appropriate, the analyses were corrected for multiple comparisons using the Bonferroni method.

### NK Cell Cytotoxicity Assays

A total of 15 healthy Chinese Southern Han subjects (six of them with *KIR AA* genotype) were recruited from Shenzhen Blood Center. PBMC were separated by density gradient from 10 ml of peripheral blood and cultured for 13–14 days using the HANK cell *in vitro* preparation kit (Hank Bioengineering, Shenzhen, China). This process uses irradiated feeder cells expressing IL15 to promote NK cell expansion as described (29) for use in immunotherapy (30, 31), and we use it here to produce NK cells for *in vitro* assay. The expansion process increases the number and activity of NK cells but does not affect KIR expression (29, 32, 33). After this process, the mean purity of CD56^+^CD3^−^ cells was 86 ± 4.5%. Antibodies used were: UCHT1 mouse anti-human CD3-FITC and B159 mouse anti-human CD56-APC (BD Biosciences, CA, USA). Target cells (1 × 10^6^) were incubated for 10 min at 37°C in 1 mL PBS containing 2.5 μmol/L CFSE and then washed in PBS. Target cells were K562, which is a leukemia derived cell line that lacks HLA class I expression, and seven primary leukemic blasts from ALL and AML patients. NK cells and target cells were combined at an effector-target-ratio of 20:1, and incubated in RPMI-1640 complete medium for 4 h at 37°C with 5% CO_2_. Dead cells were stained using 7-AAD (BD Biosciences, CA, USA) for 15 min as described (34) and measured using a BD ACCURI C6 flow cytometer (BD Biosciences, CA, USA). Spontaneous lysis of target cells was measured by including a control without NK cells, and this value was subtracted from the total percentage of specific target cell lysis. All experiments were performed in duplicate.

### Detection of CD107a Expression

A total of 18 healthy Chinese Southern Han individuals (11 of them with *KIR AA* genotype) were recruited from Shenzhen Blood Center. NK cells were isolated as above and incubated with K562 cells at a ratio of 5:1 for 5 h at 37°C. Optimal expression of CD107a on the cell surface of degranulating NK cells occurs between 4~6 h post stimulation (35). After the first 1 h, 4 μL monensin (BD Biosciences, CA, USA) was added to each 6 mL culture. The cells were then incubated with 2.5 uL human Fc block (BD Biosciences, CA, USA) at 25°C for 15 min, then stained with CD56, CD3, and CD107a mAbs. Antibodies used were: UCHT1 mouse anti-human CD3-FITC, H4A3 mouse anti-human CD107a-PECy5, and B159 mouse anti-human CD56-APC (all BD Biosciences, CA, USA). Flow cytometric analysis was performed using an ACCURI C6 machine (BD Biosciences, CA, USA) Surface expression of CD107a was assessed in CD56^+^CD3^−^ cells (36). To detect spontaneous degranulation, a control sample without target cells was included in every experiment and this information was used for background subtraction of the results.

## Results

### *KIR A* Homozygosity Protects Chinese Han From Leukemia and Non-Hodgkin's Lymphoma

**Figure 1:**
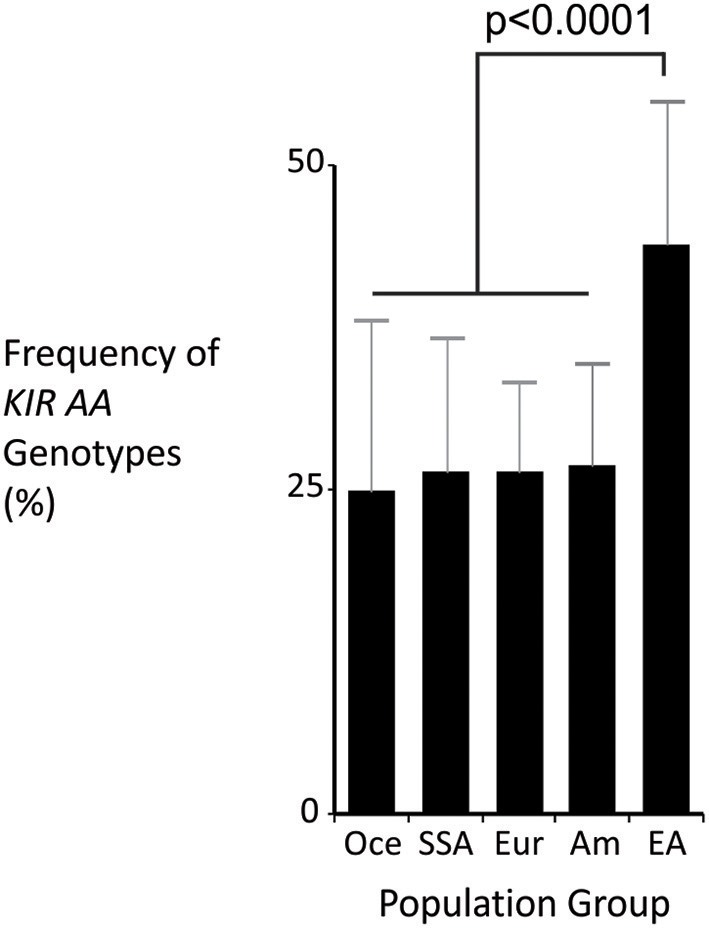
The *KIR AA* genotype is most frequent in East Asia. Mean frequencies of the *KIR AA* genotype from multiple populations representing major world groups: Oce—Oceanian (13 populations), SSA –sub-Saharan African (13 populations), Eur—European (60 populations), Am—Amerindian (24 populations), EA—East Asian (31 populations). Data were obtained from allelefrequencies.net (21). ANOVA test for between group differences *p* = 2.1 × 10^−16^. The *p*-value shown is from *t*-test of comparison of means: East Asian is significantly different to every other group, with *p* < 0.001 for each comparison. Vertical bars are S.D.

**Figure 2:**
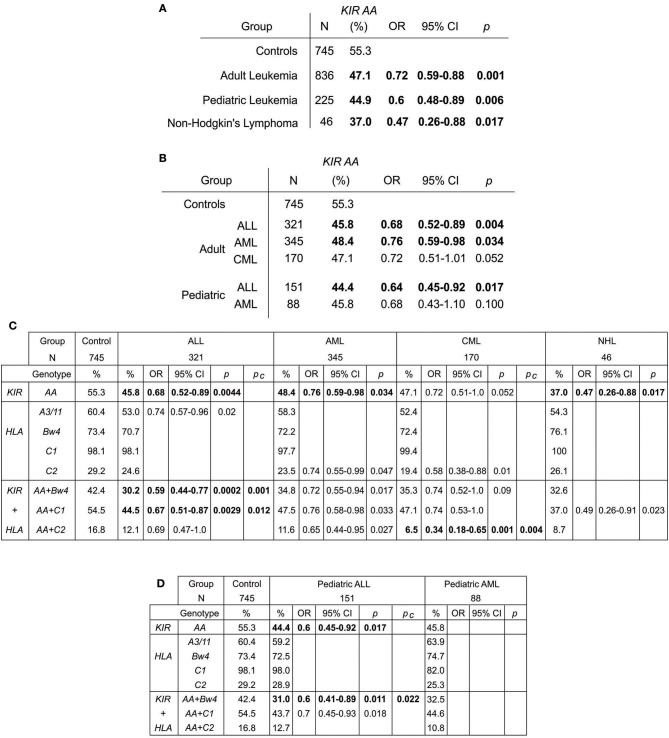
The *KIR AA* genotype associates with protection against leukemia. **(A,B)** Odds ratios and statistical significance obtained by comparing the *KIR AA* genotype frequencies of healthy controls with **(A)** adult and pediatric leukemia cases and non-Hodgkin's lymphoma, and **(B)** with the subtypes of leukemia. Statistically significant values are indicated in bold. **(C)**. Genotype frequencies, odds ratios and statistical significance obtained by comparing healthy controls (left), with three leukemia types, ALL, AML and CML (center), and NHL (right). The genotypes are: *KIR AA* genotype (top), the four KIR ligands (center) and *KIR AA* genotype in combination with each ligand (bottom). Only those results having 95% CI that do not encompass 1 are shown. P_*c*_–Bonferroni correction factors used were x4 for ligands and x3 for *ligand*+*KIR AA*. Bold indicates *p*-value remains significant after this correction. **(D)** Shows the equivalent values obtained for pediatric ALL and AML.

The *KIR A* haplotype is found in all human populations to varying frequency and is associated with NK cell function in protection from infectious diseases and in susceptibility to preeclampsia (10, 37). Because NK cells also function in tumor control, we tested whether *KIR A* affects the probability of developing leukemia. We first demonstrated that the frequency of individuals who are homozygous for the *KIR A* haplotype is significantly greater in East Asians than in other populations (*p* < 0.0001: Figure 1). We therefore examined a Chinese Southern Han cohort. In this cohort, the frequency of the *KIR AA* genotype is significantly higher among healthy individuals (55.3%) than adult leukemia patients (47.1%: OR = 0.72, 95% CI = 0.59–0.88, *p* = 0.0012) or pediatric leukemia patients (44.9%: OR = 0.6, 95% CI = 0.48–0.89, *p* = 0.006) (Figure 2A). When analyzed by disease subgroup, the *KIR AA* genotype was more frequent in healthy controls than in ALL, AML or CML patients (Figure 2B). For adult ALL (OR = 0.68, 95% CI = 0.52–0.89, *p* = 0.004) and AML (OR = 0.76, 95% CI = 0.59–0.98, *p* = 0.034), and pediatric ALL (OR = 0.64, 95% CI = 0.45–0.92, *p* = 0.017) the differences are statistically significant (Figure 2B). Similar trends were observed for CML and pediatric AML but did not reach statistical significance (*p* = 0.052 and *p* = 0.1, respectively), likely due to smaller sample sizes. We also analyzed non-Hodgkin's lymphoma (NHL) and again observed a protective effect of the *KIR AA* genotype (OR = 0.47, 95% CI = 0.26–0.88, *p* = 0.017), which was statistically significant in spite of the small sample size (*N* = 46: Figure 2A). The consistent reduction of frequency in all the disease groups analyzed strongly suggests Chinese Southern Han individuals having the *KIR AA* genotype are protected from developing leukemia or lymphoma.

### In *KIR A* Homozygotes, KIR Ligands Differentially Protect From Leukemia Subtypes

To further explore our findings, we analyzed the HLA class I ligands for KIR. *HLA class I* allele frequencies can vary significantly between the ethnic groups and geographic regions of China, for example between Northern and Southern Han (38, 39). Validating the equivalent compositions of our disease and control subjects, there were no significant differences in *HLA-A, -B* or *-C* allele or haplotype frequency spectra between controls and the full patient group (Supplemental Figures 2A,B) or from previously published frequencies of *HLA class I* alleles of Chinese Southern Han (40). Neither was there a difference between groups in the frequencies of the −21M variant of HLA-B (Supplemental Figure 2C), which can influence NK cell education (41).

KIR bind differentially to specific amino acid motifs present on non-overlapping subsets of HLA class I variants (allotypes). The HLA allotypes are HLA-A^*^03 and -A^*^11 (the A3/11 motif: KIR3DL2 ligands), HLA-A and -B allotypes that contain the Bw4 motif (KIR3DL1 ligands), HLA-B and -C allotypes containing the C1 motif (primarily KIR2DL2/3 ligands), and HLA-C allotypes containing the C2 motif (primarily KIR2DL1 ligands). We observed trends for reduction of A3/11 frequency in the adult ALL patients (60.4 vs. 53%, *p* = 0.02: Figure 2C) and reduction of C2 frequency in the adult AML (29.2 vs. 23.5%, *p* = 0.047) and CML (29.2 vs. 19.4%, *p* = 0.01) patients relative to controls. However, these findings did not remain significant after accounting for multiple testing by correction for the four ligands analyzed (Figure 2C). By contrast, combinatorial analysis of KIR and their HLA class I ligands revealed clear distinctions between the three leukemia subsets (Figures 2C,D). The *KIR AA* plus *Bw4* compound genotype was more frequent in healthy individuals (42.4%) than in subjects with ALL (30.2% adult, *p* = 0.0002, and 31.0% pediatric *p* = 0.011) or AML (34.8% adult, *p* = 0.017 and 32.5% pediatric, ns). Similarly, the *KIR AA* plus *C1* compound genotype was significantly more frequent amongst the healthy controls (54.5%) than either the ALL (44.5% adult, *p* = 0.003 and 43.7% pediatric, *p* = 0.018) or AML (47.5% adult, *p* = 0.033 and 44.6% pediatric, ns) patients. For adult ALL, the strength of the effect was consistently greater when considering *KIR* in combination with *HLA class I* than when considering *KIR* genotype alone. These associations remained significant after correction for multiple testing (Pc = 0.001 *Bw4*^+^*HLA*, and Pc = 0.012 *C1*^+^*HLA*: Figure 2C). A reduced frequency of each of the three KIR/ligand combinations was observed in AML and NHL patients, but neither association remained significant after correction, suggesting there is likely limited improvement in protection when considering ligand over that offered by KIR alone.

**Figure 3:**
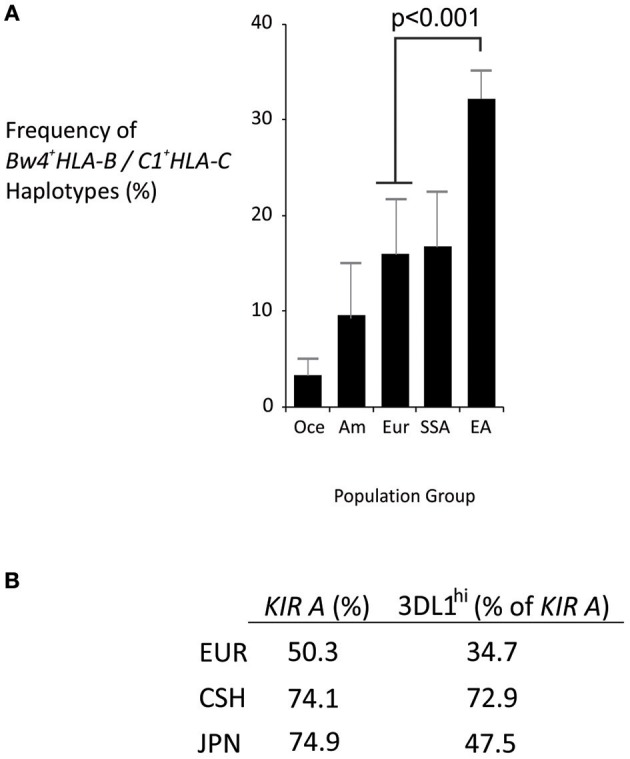
Distinctive HLA and KIR haplotypes in Chinese Southern Han. **(A)** Mean frequencies of *Bw4*^+^*HLA-B*/*C1*^+^*HLA-C* haplotypes from multiple populations representing major world groups, Oce—Oceanian (3 populations), Am—Amerindian (4 populations), Eur—European (4 populations), SSA—sub-Saharan African (6 populations), EA—East Asian (8 populations). Data were obtained from Solberg et al. (42) and haplotype frequencies calculated using the Expectation Maximization algorithm (43). Vertical bars are S.D. **(B)** Frequency of *KIR A* haplotypes in European (EUR), Chinese Southern Han (CSH) and Japanese (JPN) populations, and the percentage of *KIR A* haplotypes that express either KIR3DL1*001 or KIR3DL1*015 (KIR3DL1^hi^).

Analysis of representative world populations revealed distinctively strong linkage disequilibrium between *Bw4*^+^*HLA-B* and *C1*^+^*HLA-C* in East Asians (Figure 3A). *HLA*-B^*^46, which is the only frequent *HLA-B* allele that expresses a C1 motif, is characteristic to East Asia, common in the Chinese Southern Han (~15%), and is linked to *C1*^+^*HLA-C* (21, 40). Also in the Chinese Southern Han, all *KIR A* haplotypes express KIR2DL3^*^001, which is an inhibitory receptor that interacts with C1^+^HLA-C (26). KIR2DL3^*^001 also predominates in Japanese, another East Asian population (44). Similarly, the majority of East Asian *KIR A* haplotypes (>70%) express either KIR3DL1^*^001 or KIR3DL1^*^015, which are strongly-inhibitory KIR3DL1 allotypes specific for Bw4^+^HLA (44). Of note, the non-expressed KIR3DL1^*^004 allele that is common in Europeans (25) is absent from East Asians (44–46). Thus, in addition to the high frequency of *KIR A* haplotypes, a given Chinese Southern Han *KIR A* haplotype is more than twice as likely to express KIR3DL1^*^001 or ^*^015 than a European *KIR A* haplotype (Figure 3B). Taken together, these observations indicate that Chinese Southern Han having the *KIR AA* genotype are more likely to have multiple interactions between strongly inhibitory KIR and their corresponding HLA ligands than are European individuals, who are more commonly studied. Thus, these strongly inhibitory KIR/HLA ligand interactions may be important factors in the NK cell control of ALL and to a lesser degree AML, in Chinese Southern Han.

In contrast to ALL and AML, the strength of protection from developing CML offered by the *KIR AA* genotype was enhanced by the C2 ligand (OR = 0.34, 95% CI = 0.18–0.65, *Pc* = 0.004) to a much greater degree than by the C1/Bw4 ligands (Figure 2C). Although the sample size of the CML group is relatively small, the strength of the effect provides compelling evidence that Chinese Southern Han individuals having the *KIR AA* plus *C2*^+^*HLA-C* genotype are less likely to develop CML than those who do not have this compound genotype. KIR2DL1, which is encoded by the *KIR A* haplotype, has high specificity for C2^+^HLA-C, but the strength of inhibition resulting from the interaction varies across KIR2DL1 subtypes (47, 48). In East Asians, only two KIR2DL1 subtypes predominate: strongly inhibitory KIR2DL1^*^003 encoded by *KIR A* haplotypes, and weakly inhibitory KIR2DL1^*^004 encoded by *KIR B* haplotypes (44) (and ZD, PJN unpublished). NK cells expressing KIR2DL1^*^003 are highly effective at killing cells that have reduced C2^+^HLA-C expression, whereas those expressing KIR2DL1^*^004 are significantly weaker in this regard (47, 48). Thus, individuals who have the *KIR AA* genotype in this population likely have two genomic copies of the strongly inhibiting KIR2DL1 allotype, KIR2DL1^*^003, and should be best equipped at recognizing and eliminating tumor cells having aberrant C2^+^HLA-C expression. This could take the form of reduction or loss of HLA expression (49, 50) or presentation of tumor neoantigen peptides by the C2^+^HLA-C molecules, abrogating interaction with KIR2DL1 (5, 51). This result thus identifies a role for strongly functional KIR2DL1 allotypes in protection from CML in Chinese Han. This finding strikes strong parallel with several reproductive diseases including preeclampsia, where the *KIR AA* genotype in combination with C2 ligand impacts the risk of developing the disease (37), and highlights the need for study of populations in addition to Europeans for understanding mechanisms of protection from disease (52).

In summary, the *KIR AA* genotype confers differential protection against ALL, AML, CML and NHL; whereas the C2 ligand enhances *KIR AA* protection against CML, the C1 and Bw4 ligands enhance *KIR AA* protection against ALL, and the *KIR AA* genotype alone protects from AML and NHL.

### NK Cells From *KIR A* Homozygous Individuals Are Strongly Cytotoxic to Leukemia Cells

**Figure 4:**
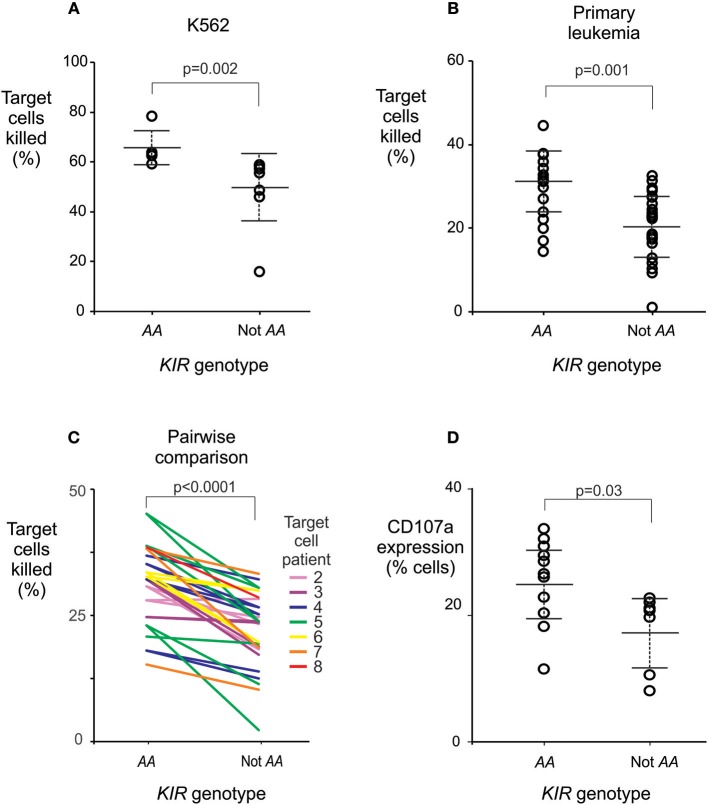
The *KIR AA* genotype correlates with enhanced potential of NK cells to lyse leukemic cells. **(A)** Mean % killing of K562 target cells by NK cells isolated from six healthy individuals with (left) and nine without (right) the *KIR AA* genotype. Each dot represents the mean result from a duplicated assay (Supplemental Figure 3E). *P*-value is from a Mann-Whitney *U*-test. **(B)** Mean % killing of primary leukemia cells using the same NK cells as **(A)**. The primary leukemia targets were obtained from two ALL patients and five AML patients (Supplemental Figure 3). Each dot represents the mean result from a duplicated assay (Supplemental Figure 3). *P*-value is from a Mann-Whitney *U*-test. Six NK cell donors were tested against two targets, seven were tested against three targets, and two against four targets, as detailed in Supplemental Figure 3. **(C)** Pairwise comparisons of % killing by NK cells from *KIR AA* vs. non *AA* individuals against identical target leukemia cells. Each line represents one of the seven targets, incubated with NK cells from an *AA*^+^ donor (left) or an *AA*^−^ donor (right), at the same time and conditions. Line colors indicate the seven individual targets as given in Supplemental Figure 3 (Target 1 is K562; not shown for clarity). *P*-value is from a paired *t*-test. **(D)** Shows CD107a expression by NK cells following incubation with K562 targets. The NK cells were isolated from a further 11 healthy individuals with (left) and seven without (right) the *KIR AA* genotype. *P*-value is from a Mann-Whitney *U*-test. Error bars are mean ± sd. The NK killing assays are described in Supplemental Figure 3.

To investigate whether individuals who are *KIR AA*^+^ have stronger responses to leukemia than other individuals do, we measured the cytotoxicity against leukemia cells, of NK cells from healthy individuals with and without *KIR AA*. We used standard assays for NK cell activity (30, 31, 35, 36) to study six healthy Chinese Southern Han subjects having the *KIR AA* genotype, and nine without it. NK cells from individuals with the *KIR AA* genotype demonstrated significantly greater cytotoxicity against the HLA class I negative K562 leukemia cell line, a standard target used to measure NK cell cytotoxicity, than NK cells isolated from individuals without the *AA* genotype (mean killing 65 vs. 49%, *p* = 0.002; Figure 4A). These data suggest that NK cells from *KIR AA* individuals are better educated to respond to loss of HLA class I expression than are those from individuals carrying other *KIR* genotypes.

Next, the same NK cells were tested for their cytotoxicity against primary leukemic cells, which were isolated from five patients with AML and two with ALL (Supplemental Figure 3). Again, NK cells isolated from individuals who have the *KIR AA* genotype had a stronger cytotoxic response than those isolated from non-*AA* individuals (29.9 vs. 20.5%, *p* = 0.001; Figure 4B). In line with previous observations on European subjects (53), we observed variability in the baseline killing of leukemia cells across NK cell donors. This may be due to differential education by donor HLA class I allotypes. Although all the donors and patients studied possess the C1 ligand (Supplemental Figure 3), the very high number of subjects required precludes from completely controlling for HLA variation. Nevertheless, a higher degree of cytotoxicity was consistently observed for NK cells from *KIR A*A^+^ donors as compared to *KIR A*A^−^ donors regardless of the source of leukemia cell targets (*p* < 0.0001 by paired *t*-test; Figure 4C). This result was further validated in an independent set of 18 healthy individuals and a complementary method, which measured the cell surface expression of CD107a that accompanies degranulation of NK cells (35). Here, a mean of 25.4 % NK cells from *KIRAA*^+^ subjects expressed CD107a following contact with K562 cells, vs. 17.8% from non-*AA* subjects (*p* = 0.03: Figure 4D). In summary, these findings show that in Chinese Southern Han, NK cells from individuals with the *KIR AA* genotype exert greater cytotoxicity against leukemic cells than those from individuals having any other *KIR* genotype.

## Discussion

KIR are highly polymorphic and the distribution of *KIR* haplotypes differs markedly across human populations (11). Because multiple previous studies were consistent with, but had not identified, a role for the *KIR AA* genotype in protection from leukemia, we studied a cohort of Chinese Southern Han from East Asia, where the *KIR A* haplotype is at highest frequency. We showed that possession of the *KIR AA* genotype protects individuals from developing either of three types of leukemia and one lymphoma analyzed. These results were further refined by considering the HLA ligands for KIR in the analysis. Importantly, *in vitro* functional assays demonstrated that NK cells from healthy individuals with *KIR AA* genotype had strong reactivity against leukemic cells.

Because there have been conflicting reports on the role of KIR in protection from leukemia, we sought to study a large number of patients and controls that were matched by clearly defined age and ethnicity. We studied over 1,000 patients and 745 controls and observed no significant difference in the overall frequency distribution of *HLA class I* alleles between the two groups or compared with previous studies of Chinese Southern Han (40). Because allele frequency spectra of these highly polymorphic genes characterize and distinguish human populations (42), this finding validates the self-defined ancestry of the cohorts as well as their suitability for use in these comparisons. We studied subjects from China, where the mean age for leukemia diagnosis is younger than that seen for Europeans (54–56), and compared them with healthy blood donors having a similar age distribution. The high frequency of *KIR AA* homozygous individuals observed in our control group is consistent with multiple previous studies of healthy Chinese Southern Han (19, 57–59). We also observed a difference in *KIR AA* frequency between AML patients and controls of similar magnitude as that seen in a previous study of Chinese subjects from Singapore (19). One caveat is that we studied patients selected as potential transplantation recipients, but we consider it unlikely that *KIR/HLA* genotype affects this initial decision. Moreover, our finding that possession of the *KIR AA* genotype consistently protects from at least three types of leukemia and one lymphoma was further validated by *in vitro* functional assays of NK cell cytotoxicity and lytic granule release.

Our results suggest that any contradictory findings in the association of KIR/HLA and leukemia that have been reported are likely to be driven by the relative population frequencies of the *KIR A* haplotypes and the KIR allotypes they encode. Many previous studies are concordant with the results presented here (14, 15, 17–19, 60), while others that differ could be explained by cryptic differences in ancestries of patients and controls, resulting in differences in *KIR A* haplotype frequencies, and the alleles present on those haplotypes (11, 26, 61–65). We hypothesize that the strongly inhibitory KIR allotypes that characterize East Asian *KIR* haplotypes efficiently educate NK cells to detect changes in HLA expression that can occur in leukemic cells (20, 44). These changes may include loss or reduction of HLA expression, or alterations to the peptide repertoire that may be detected by NK cells through KIR binding (5, 51). That the same strongly-inhibiting KIR allotypes are present in other populations, albeit to lower frequencies (11), suggests their effect on leukemia control will be similar, but much larger sample sizes will be required to detect them. We studied a population with relatively low genetic diversity, such that possession of the *KIR A* haplotype is accompanied by a uniform set of alleles of the component genes. High resolution analyses will likely be required to account for the substantial allelic diversity of any further population groups analyzed (66).

Although the *KIR AA* genotype appears to protect against leukemia and NHL in Chinese Southern Hans, there were differences in the HLA ligand required to strengthen this effect. These differences may be due to the distinct maturation stages and the non-overlapping subsets of lineage markers and fusion proteins expressed by the leukemia subtypes (67, 68). The clearest ligand effect was observed for CML, which is likely due to the relative uniformity of this leukemia class (69). The least defined ligand effect was observed for AML, likely due to the highly heterogeneous nature of this disease (70). Another ligand for inhibitory NK cell receptors is HLA-E, which can present peptide fragments derived from the expression of HLA-A, -B or -C. CD94/NKG2A educates and modulates NK cells through binding HLA-E, complementing the role of KIR and extending the capacity of NK cells to monitor HLA class I expression (41, 71). Although CD94/NKG2A is conserved, diversity across individuals occurs because only some HLA-B alleles encode the −21M variant peptide that binds to HLA-E. Accordingly, a recent study showed −21M HLA-B associated with increased survival for AML patients receiving immune therapy (72). However, as we observed no significant differences in HLA allele frequencies (Supplemental Figure 2), there were no significant differences in frequency of the HLA-B −21M variant between the patients and controls of our study. This indicates that HLA-B −21M polymorphism is unlikely to affect leukemia susceptibility in Chinese Southern Han.

Although the endpoint of our analysis was disease diagnosis, a limitation is that we were not able to perform functional tests of NK cells extracted from leukemia patients, or phenotypic analyses of HLA expression by leukemic cells. The exception is the K562 target, which is a leukemia cell known to lack HLA class I expression. Another limitation is that the relatively low sample numbers we obtained for the functional tests did not allow for thorough analysis of KIR ligand mismatches between NK cell donor and leukemia target cells. It will be important in future work to examine how changes in HLA expression or neo-antigen peptide presentation may affect the NK cell response to cells that could be leukemogenic, and ultimately how they may influence disease course. These kinds of experiments require pre-genotyping of large numbers of individuals, and will be greatly facilitated by involving populations where the respective genotypes are at high frequency, including the Chinese Southern Han.

In conclusion, this study enhances our understanding of the immune response to leukemia in the world's largest population group, shows the benefit of including specific well-defined populations in study of diseases involving highly polymorphic genes, and identifies a role for NK cell education by inhibitory KIR in control of leukemia. In light of recent studies that promote expansion beyond the usual European populations analyzed (73), the benefits of studying well-defined population groups is becoming increasingly apparent.

## Data Availability

All datasets generated for this study are included in the manuscript and/or the Supplementary Files.

## Ethics Statement

The study was approved by the ethics review board of Shenzhen Blood Center, Shenzhen, Guangdong, China. Written informed consent was obtained from all study participants.

## Author Contributions

ZD, SC, JZha, WH, MC, and PN: experiment and analysis design. ZD, YQ, RC, JZhu, JZhe, MZ, GZ, LH, HZ, LL, and WZ: data generation and interpretation. ZD, YQ, QY, MM, XG, and PN: Data analysis. ZD, JZha, XG, MM, MC, and PN: Manuscript preparation. All authors: manuscript review and approval.

### Conflict of Interest Statement

MZ is an employee of Shenzhen Hank Bioengineering Institute. The remaining authors declare that the research was conducted in the absence of any commercial or financial relationships that could be construed as a potential conflict of interest.
