# Supplementary material for: Natural Killer Cells Offer Differential Protection From Leukemia in Chinese Southern Han
Source: Front Immunol. 2019 Jul 16;10:1646. doi: 10.3389/fimmu.2019.01646 (PMC6646668; doi:10.3389/fimmu.2019.01646)
Supplement: Supplementary file 1 [file Data_Sheet_1.pdf]

Supplementary Material

Supplemental Figure 1. Characteristics of Chinese Southern Han leukemia patients and controls.

Supplemental Figure 2. ***HLA class I*** **frequencies of patients and controls**

A. *HLA-A*, *-B* and *-C* frequencies of control (left) and patient (right) samples. There is no statistically significant difference between controls and patients for either locus (pairwise t-test, all p>0.99). The full leukemia patient set was used for this comparison (N=1061).

B. Frequencies for the ten most frequent *HLA class I* haplotypes in each group.

C. Allele frequencies for HLA-B -21M in control and patient groups.

Supplemental Figure 3. Details of NK cytotoxicity assay

A-D. Representative FACS plots of the cytotoxic assay. (A) K562 target cells only, and K562 following incubation with NK cells from an individual having (B) *KIR AA* genotype (C) *AB4* genotype and (D) *BB117* genotype. CFSE+ 7AAD+ cells are the dead targets. The results correspond to Experiment 1 from panel E.

E. *KIR* genotypes of healthy donors from whom NK cells were extracted, and the results of cytotoxicity assays using leukemia cells as targets are shown. (Left) KIR genotypes are named according to allelefrequencies.net [23]. (Right) Target cells were (1) K562, and (2-8) leukemia cells obtained from patients at diagnosis (ALL or AML). The leukemic cells were obtained from bone marrow (BM) or PBMC. The numbers show the percentage of target cells that were killed. All experiments were performed in duplicate. All 15 of the NK-cell-donors were tested against the K562 cell line. As the leukemia cells were limited in quantity, it was not possible to test every donor NK cells against every leukemia target. Spontaneous lysis of target cells (e.g. Panel A) was measured for each target cell for each experiment and subtracted from the output to give the results shown.

F-I. Representative FACS plots of the CD107a assay. (F-G) CD3-CD56+ cells unstimulated. (H-I) CD3-CD56+ cells from the same donor incubated for 5 hours with K562 cells.

Adult Leukemia

	Controls	ALL	AML	CML
N	745	321	345	170
Female (%)	15.3	40.6	43.4	27.3
Male (%)	84.7	59.4	56.6	72.7
Mean age	32.7	29.8	32.9	31.4
range	18-55	18-57	18-57	18-58

Pediatric Leukemia

ALL	AML
142	83
40.4	43.2
59.6	56.8
10.9	12.25
1-17	1-17

AML Classification by FAB (total)

M0	M1	M2	M3	M4	M5	M6	M7	Unknown
0	23	117	4	71	106	16	3	93

Supplemental Figure 1

A

HLA-A	Allele Frequency (%) Control	Allele Frequency (%) Patient
*01:01	1.75	0.74
*01:03	0.13	
*02:01	8.05	7.58
*02:03	5.30	8.46
*02:05	0.20	0.14
*02:06	2.55	3.72
*02:07	12.01	14.29
*02:09		0.05
*02:10	0.13	0.14
*02:17	0.07	
*02:22		0.05
*02:53N		0.05
*03:01	1.34	0.51
*03:02	0.13	0.09
*11:01	30.34	30.15
*11:02	3.02	2.99
*11:03	0.07	
*11:04	0.07	
*11:06	0.00	0.05
*11:18	0.07	
*11:36	0.07	
*23:01	0.20	0.14
*24:02	15.84	15.21
*24:03	0.07	0.14
*24:07	0.20	0.78
*24:10	0.07	0.14
*24:20		0.05
*24:64		0.05
*26:01	2.28	1.38
*29:01	0.67	0.32
*29:02	0.07	
*30:01	2.62	1.70
*30:04		0.05
*30:07		0.05
*31:01	2.82	1.52
*31:02		0.05
*32:01	0.87	0.18
*33:01	0.20	
*33:03	8.26	8.73
*34:01	0.07	
*68:01	0.34	0.28
*74:01	0.07	
*74:02	0.07	0.28

HLA-B	Allele Frequency (%) Control	Allele Frequency (%) Patient
*07:02	1.14	0.37
*07:05	0.67	0.32
*07:06		0.09
*07:10	0.07	0.05
*08:01	0.60	0.37
*13:01	7.92	8.36
*13:02	2.68	1.93
*14:01		0.05
*14:02	0.13	
*15:01	3.62	3.31
*15:02	5.37	6.53
*15:05	0.20	0.09
*15:07	0.27	0.14
*15:11	1.01	0.78
*15:12	0.40	0.28
*15:18	0.60	0.51
*15:19	0.13	0.09
*15:21	0.13	
*15:25	0.60	0.97
*15:27	0.27	0.51
*15:32	0.07	0.18
*15:58	0.07	
*18:01	0.40	0.14
*18:02		0.18
*27:04	1.81	1.29
*27:05	0.20	0.28
*27:06	0.20	0.05
*27:07	0.20	
*35:01	2.35	1.56
*35:02	0.07	0.09
*35:03	0.54	0.32
*35:05	0.34	1.06
*35:08	0.07	
*37:01	0.87	0.51
*38:01	0.27	0.14
*38:02	3.15	5.06
*39:01	1.75	1.52
*39:05	0.13	0.14
*39:09		0.05
*40:01	15.91	16.87
*40:02	1.14	1.19
*40:03	0.07	0.18
*40:06	2.08	0.92

HLA-B	Allele Frequency (%) Control	Allele Frequency (%) Patient
*40:10	0.07	
*40:40	0.07	
*40:55		0.05
*41:01	0.07	
*41:02	0.07	
*44:02	0.34	0.28
*44:03	0.87	1.10
*44:07	0.07	
*45:01	0.07	0.05
*46:01	14.16	17.19
*48:01	1.07	0.64
*48:03	0.34	0.51
*49:01	0.07	
*50:01	0.20	0.09
*51:01	4.30	4.64
*51:02	1.48	1.29
*51:06		0.05
*51:07		0.05
*52:01	1.41	0.78
*54:01	3.42	3.17
*55:01	0.07	0.05
*55:02	3.36	3.54
*55:04		0.09
*55:12	0.07	
*56:01	1.14	0.97
*56:03		0.28
*56:04	0.07	0.28
*56:10		0.05
*57:01	0.67	0.18
*58:01	8.32	8.09
*58:02		0.05
*59:01	0.07	
*67:01	0.47	0.09
*81:02	0.20	

HLA-C	Allele Frequency (%) Control	Allele Frequency (%) Patient
*01:02	19.26	22.24
*01:03	0.34	0.46
*01:08	0.07	0.14
*02:02	0.27	0.23
*03:01	0.07	
*03:02	8.26	8.23
*03:03	4.90	3.91
*03:04	13.02	13.88
*03:17	0.07	0.05
*03:36		0.05
*03:38	0.07	
*04:01	5.10	4.55
*04:03	1.54	1.75
*04:06	0.07	0.09
*04:107		0.05
*04:15	0.07	
*05:01	0.34	0.14
*06:02	4.30	2.80
*07:01	0.27	
*07:02	18.26	19.53
*07:04	0.67	0.60
*07:06	0.20	0.69
*07:19	0.20	
*07:43	0.07	
*07:66		0.05
*07:67		0.05
*08:01	8.93	9.05
*08:02	0.13	0.05
*08:03	0.40	0.14
*12:02	2.68	2.16
*12:03	2.42	1.33
*14:02	3.62	4.23
*14:03	0.34	0.28
*15:02	3.15	2.80
*15:04		0.05
*15:05	0.60	0.32
*16:02	0.20	0.09
*16:04		0.05
*17:01	0.13	

B

Controls

HLA-A	HLA-B	HLA-C	Haplotype frequency (%)
*02:07	*46:01	*01:02	8.28
*33:03	*58:01	*03:02	6.43
*11:01	*40:01	*07:02	5.19
*11:01	*13:01	*03:04	4.40
*11:01	*15:02	*08:01	3.30
*11:01	*46:01	*01:02	2.37
*30:01	*13:02	*06:02	2.21
*24:02	*40:01	*03:04	1.91
*24:02	*40:01	*07:02	1.71
*02:03	*38:02	*07:02	1.46
*11:01	*40:01	*03:04	1.27

Patients

HLA-A	HLA-B	HLA-C	Haplotype frequency (%)
*02:07	*46:01	*01:02	10.11
*33:03	*58:01	*03:02	6.82
*11:01	*40:01	*07:02	5.21
*11:01	*15:02	*08:01	4.61
*11:01	*13:01	*03:04	3.95
*02:03	*38:02	*07:02	3.19
*11:01	*46:01	*01:02	2.63
*24:02	*40:01	*03:04	2.12
*11:01	*51:01	*14:02	1.77
*24:02	*40:01	*07:02	1.57
*30:01	*13:02	*06:02	1.52

C

	HLA-B -21M Allele Freq. (%)
Controls	10.00
Patients	9.38
ALL	9.81
AML	9.46
CML	8.43

Supplemental Figure 2

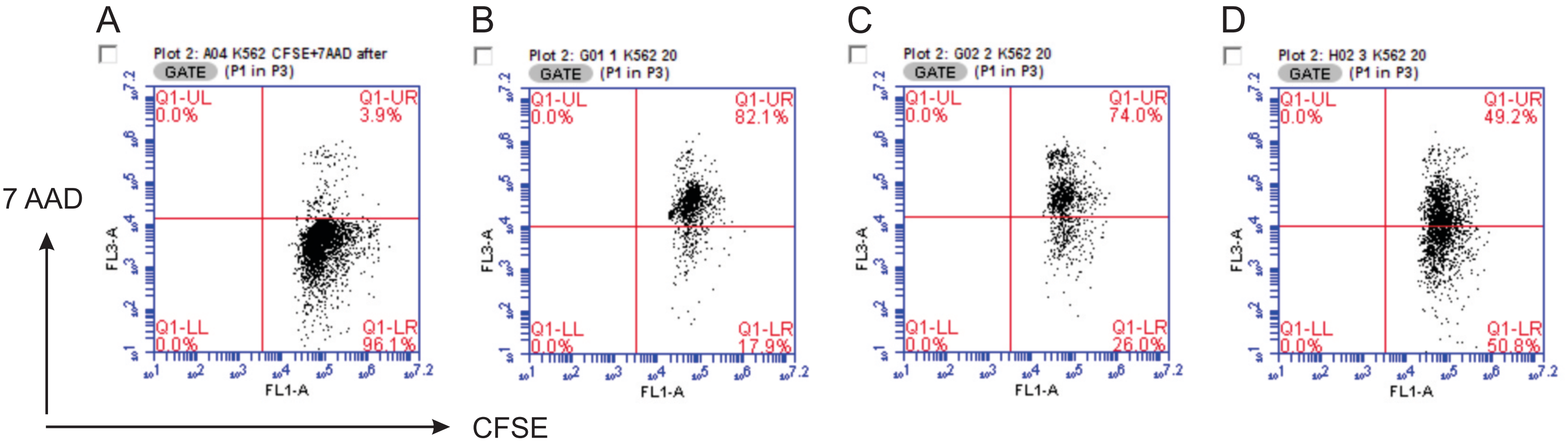

E

	NK cells						Target leukemia cells																								
Experiment	Donor	KIR ligand C1	C2	Bw4	A3/11	*KIR* Genotype	Patient	1			2			3			4			5			6			7			8		
							Diagnosis	K562 cell line			ALL			AML-M3			AML-M5			AML-M4			AML-M1			AML			ALL		
							Source	-			BM			BM			PBMC			BM			PBMC			PBMC			BM		
							C1	-			+			+			+			+			+			+			+		
							C2	-			-			+			-			-			-			-			+		
							Bw4	-			+			+			-			-			+			-			+		
							A3/11	-			+			-			+			+			-			+			-		
								1	2	x̄	1	2	x̄	1	2	x̄	1	2	x̄	1	2	x̄	1	2	x̄	1	2	x̄	1	2	x̄
1	1	+	-	-	+	AA1		78.2	78.4	78.3	32.4	21.6	27.0	21.2	26.4	23.8															
	2	+	-	-	+	AB4		70.1	46.0	58.0	25.3	29.6	27.4	20.9	24.6	22.8															
	3	+	-	+	-	BB117		45.3	46.5	45.9	20.4	26.9	23.7	25.0	20.2	22.6															
2	4	+	-	-	-	AA1		58.9	65.6	62.3	31.3	28.3	29.8	32.8	29.5	31.2															
	5	+	-	-	+	AB6		59.3	57.8	58.6	15.8	19.0	17.4	18.8	16.8	17.8															
	6	+	+	+	-	AB4		60.6	53.6	57.1	20.5	24.1	22.3	17.4	15.2	16.3															
3	7	+	-	+	+	AA1		71.5	46.5	59.0							39.5	29.1	34.3	43.4	45.5	44.4	31.9	33.3	32.6						
	8	+	-	-	+	AA1		67.5	59.8	63.6							32.9	29.7	31.3	39.6	36.1	37.8	31.5	32.4	31.9						
	9	+	+	+	-	BB69		45.3	46.5	48.5							26.3	25.1	25.7	24.3	21.5	22.9	18.9	18.3	18.6						
	10	+	+	+	+	BB70		60.1	57.4	58.8							23.2	25.6	24.4	32.9	26.0	29.5	28.4	29.4	28.9						
4	11	+	-	+	-	AA1		66.9	59.5	63.2							21.3	12.7	17.0	27.5	16.7	22.1				36.9	38.1	37.5			
	12	+	-	-	+	AB2		42.1	49.4	45.7							13.8	11.7	12.8	15.7	4.8	10.3				31.2	33.6	32.4			
	13	+	-	-	+	BB76		19.7	12.3	16.0							13.7	9.4	11.5	0.7	1.3	1.0				17.2	18.1	17.6			
5	14	+	-	-	+	AA1		62.3	63.4	62.9							36.5	35.2	35.9	20.6	19.2	19.9				13.9	14.7	14.3	38.6	36.7	37.7
	15	+	+	+	-	AB3		53.1	57.6	55.3							32.5	30.2	31.3	18.3	18.5	18.4				9.4	9.2	9.3	27.0	28.3	27.6

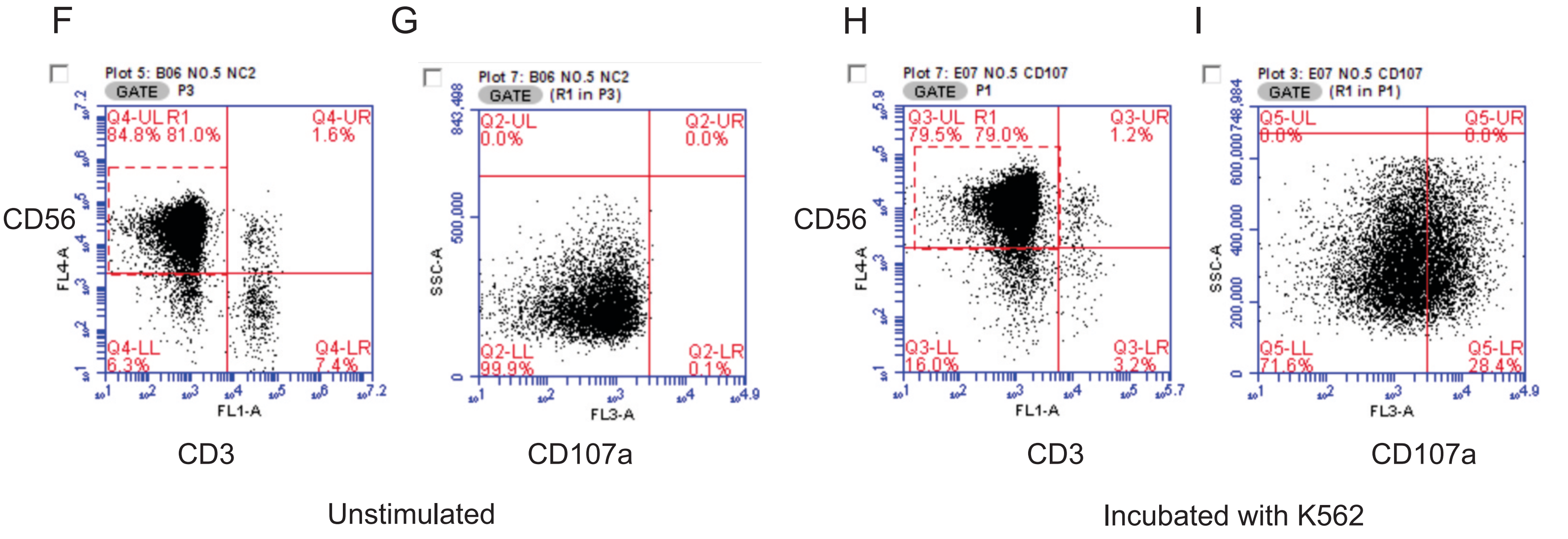

Supplemental Figure 3
